# 5-(Pyridin-4-ylmeth­yl)-1*H*-pyrazolo­[3,4-*d*]pyrimidin-4(5*H*)-one

**DOI:** 10.1107/S1600536811025025

**Published:** 2011-07-06

**Authors:** Abdulsalam Alsubari, Youssef Ramli, El Mokhtar Essassi, Hafid Zouihri

**Affiliations:** a Laboratoire de Chimie Organique Hétérocyclique, Pôle de Compétences Pharmacochimie, Université Mohammed V-Agdal, BP 1014 Avenue Ibn Batout, Rabat, Morocco; b Laboratoires de Diffraction des Rayons X, Centre National pour la Recherche, Scientifique et Technique, Rabat, Morocco

## Abstract

In the title compound, C_11_H_9_N_5_O, the pyrazolo­pyrimidin-4-one ring system is almost planar, with a maximum deviation of 0.0546 (13) Å for the O atom. The crystal packing is stabilized by inter­molecular N—H⋯N, C—H⋯O and C—H⋯N hydrogen bonds. In addition, π–π stacking is found between the pyridine ring and the pyrazolo­pyrimidin-4-one ring systems, with centroid–centroid distances in the range 3.9627 (12)–4.6781 (12) Å.

## Related literature

For a related structure, see: Al Subari *et al.* (2010 ▶). For the biological activity of pyrazolo­pyrimidinone derivatives, see: Kim *et al.* (2001 ▶); Ali *et al.* (2009 ▶).
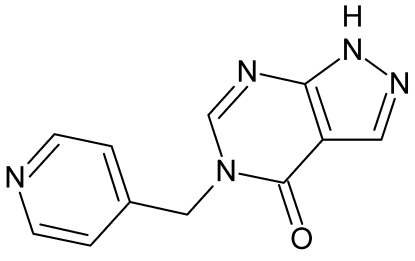


## Experimental

### 

#### Crystal data

C_11_H_9_N_5_O
*M* *_r_* = 227.23
Monoclinic, 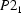

*a* = 4.6371 (3) Å
*b* = 19.2731 (10) Å
*c* = 5.8593 (3) Å
β = 102.498 (2)°
*V* = 511.24 (5) Å^3^
*Z* = 2
Mo *K*α radiation
μ = 0.10 mm^−1^
*T* = 296 K
0.25 × 0.22 × 0.17 mm

#### Data collection

Bruker APEXII CCD detector diffractometer
6285 measured reflections
2603 independent reflections
2201 reflections with *I* > 2σ(*I*)
*R*_int_ = 0.030

#### Refinement

*R*[*F* ^2^ > 2σ(*F* ^2^)] = 0.036
*wR*(*F* ^2^) = 0.085
*S* = 1.05
2603 reflections
158 parameters
2 restraints
H atoms treated by a mixture of independent and constrained refinement
Δρ_max_ = 0.16 e Å^−3^
Δρ_min_ = −0.22 e Å^−3^

### 

Data collection: *APEX2* (Bruker, 2005 ▶); cell refinement: *SAINT* (Bruker, 2005 ▶); data reduction: *SAINT*; program(s) used to solve structure: *SHELXS97* (Sheldrick, 2008 ▶); program(s) used to refine structure: *SHELXL97* (Sheldrick, 2008 ▶); molecular graphics: *PLATON* (Spek, 2009 ▶); software used to prepare material for publication: *publCIF* (Westrip, 2010 ▶).

## Supplementary Material

Crystal structure: contains datablock(s) I, global. DOI: 10.1107/S1600536811025025/fj2436sup1.cif

Structure factors: contains datablock(s) I. DOI: 10.1107/S1600536811025025/fj2436Isup2.hkl

Supplementary material file. DOI: 10.1107/S1600536811025025/fj2436Isup3.cml

Additional supplementary materials:  crystallographic information; 3D view; checkCIF report

## Figures and Tables

**Table 1:** Hydrogen-bond geometry (Å, °)

*D*—H⋯*A*	*D*—H	H⋯*A*	*D*⋯*A*	*D*—H⋯*A*
N3—H3*N*⋯N5^i^	0.90 (2)	1.96 (2)	2.840 (2)	168 (2)
C4—H4⋯N4^ii^	0.93	2.61	3.526 (2)	167
C9—H9⋯N2^iii^	0.93	2.36	3.289 (2)	174
C11—H11⋯O1^iv^	0.93	2.52	3.430 (2)	167

Symmetry codes: (i) 
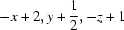
; (ii) 
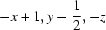
; (iii) 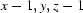; (iv) 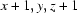.

**Fig. 1.**
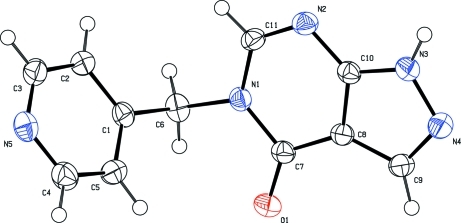
Molecular view of the title compound showing the atom-labeling scheme. Displacement ellipsoids are drawn at the 30% probability level. H atoms are represented as small spheres of arbitrary radii.

**Fig. 2.**
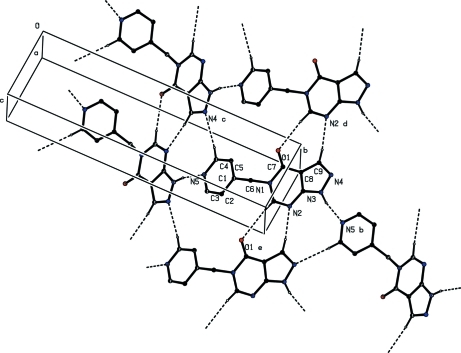
Partial packing view showing the chain formed by N—H···O and C—H···N hydrogen bondings. H atoms not involved in hydrogen bonds have been omitted for clarity.

## supplementary crystallographic information

### Comment

Pyrazolopyrimidinone derivatives have attracted the attention of numerous researchers over many years due to their important biological activities [Kim, *et al.* 2001 and Ali, *et al.* 2009].

In the title compound, C_11_H_9_N_5_O, the 4*H*-pyrazolo[3,4-*d*]pyrimidin-4-one core is almost planar (maximum atomic deviation = 0.0546 (13) Å for the oxygen atom of the system) and makes a dihedral angle of 73.94 (7)° with the attached pyridin ring (maximum atomic deviation = 0.041 (18) Å of the nitrogen atom of the ring). The crystal packing is stabilized by N—H···O and C—H···N intermolecular H-bonds and π–π stacking between pyridin and pyrazolo ring systems [*Cg* to *Cg* distances = 4.6781 (12) Å to 3.9627 (12) Å].

### Experimental

allopurinol (1 g, 7.4 mmol), 4-chloromethylpyridine (1.8 g, 14.7 mmol) and potassium carbonate (1.5 g, 11.2 mmol) with amount of catalytic tetra-*n*-butylammonium bromide were stirred in DMF (30 ml) for 72 h. The solid material was removed by filtration and the solvent evaporated under vacuum. Dichloromethane (20 ml) was added and the solution filtered. The solid product was purified by recrystallization from ethanol to afford white crystals in 60% yield.

### Refinement

The H atoms bound to C were treated as riding with their parent atoms [C—H distances are 0.93Å for CH groups with *U*_iso_(H) = 1.2 *U*_eq_(C), and 0.97 Å for CH3 groups with *U*_iso_(H) = 1.5 *U*_eq_(C). The nitrogen-bound H atoms were located in a difference Fourier map, and were refined with distance restraints of N–H 0.88 (2).

The title compound crystallizes in the non centrosymmetric space group *P*2_1_ and as the absolute configuration is not determined from the measured data, the Friedel equivalent reflections are merged before refinement with *XPREP* software.

### Crystal data

**Table:**

C_11_H_9_N_5_O	*F*(000) = 236
*M**_r_* = 227.23	*D*_x_ = 1.476 Mg m^−^^3^
Monoclinic, *P*2_1_	Mo *K*α radiation, λ = 0.71073 Å
Hall symbol: P 2yb	Cell parameters from 342 reflections
*a* = 4.6371 (3) Å	θ = 2.4–25.6°
*b* = 19.2731 (10) Å	µ = 0.10 mm^−^^1^
*c* = 5.8593 (3) Å	*T* = 296 K
β = 102.498 (2)°	Prism, colourless
*V* = 511.24 (5) Å^3^	0.25 × 0.22 × 0.17 mm
*Z* = 2	

### Data collection

**Table:**

Bruker APEXII CCD detector diffractometer	2201 reflections with *I* > 2σ(*I*)
Radiation source: fine-focus sealed tube	*R*_int_ = 0.030
graphite	θ_max_ = 28.9°, θ_min_ = 2.1°
ω and φ scans	*h* = −6→6
6285 measured reflections	*k* = −26→26
2603 independent reflections	*l* = −7→7

### Refinement

**Table:**

Refinement on *F*^2^	Primary atom site location: structure-invariant direct methods
Least-squares matrix: full	Secondary atom site location: difference Fourier map
*R*[*F*^2^ > 2σ(*F*^2^)] = 0.036	Hydrogen site location: inferred from neighbouring sites
*wR*(*F*^2^) = 0.085	H atoms treated by a mixture of independent and constrained refinement
*S* = 1.05	*w* = 1/[σ^2^(*F*_o_^2^) + (0.0427*P*)^2^ + 0.0014*P*] where *P* = (*F*_o_^2^ + 2*F*_c_^2^)/3
2603 reflections	(Δ/σ)_max_ = 0.001
158 parameters	Δρ_max_ = 0.16 e Å^−^^3^
2 restraints	Δρ_min_ = −0.22 e Å^−^^3^

### Special details

**Table:**

Geometry. All e.s.d.'s (except the e.s.d. in the dihedral angle between two l.s. planes) are estimated using the full covariance matrix. The cell e.s.d.'s are taken into account individually in the estimation of e.s.d.'s in distances, angles and torsion angles; correlations between e.s.d.'s in cell parameters are only used when they are defined by crystal symmetry. An approximate (isotropic) treatment of cell e.s.d.'s is used for estimating e.s.d.'s involving l.s. planes.
Refinement. Refinement of *F*^2^ against ALL reflections. The weighted *R*-factor *wR* and goodness of fit *S* are based on *F*^2^, conventional *R*-factors *R* are based on *F*, with *F* set to zero for negative *F*^2^. The threshold expression of *F*^2^ > σ(*F*^2^) is used only for calculating *R*-factors(gt) *etc*. and is not relevant to the choice of reflections for refinement. *R*-factors based on *F*^2^ are statistically about twice as large as those based on *F*, and *R*- factors based on ALL data will be even larger.

### Fractional atomic coordinates and isotropic or equivalent isotropic displacement parameters (Å^2^)

**Table:**

	*x*	*y*	*z*	*U*_iso_*/*U*_eq_	
N2	0.7680 (3)	1.03978 (7)	0.5859 (2)	0.0280 (3)	
N1	0.3899 (3)	0.95388 (6)	0.5329 (2)	0.0242 (3)	
N3	0.7129 (3)	1.11511 (7)	0.2529 (2)	0.0275 (3)	
N5	0.8324 (3)	0.71619 (7)	0.6387 (3)	0.0326 (3)	
N4	0.5292 (3)	1.12382 (7)	0.0394 (2)	0.0312 (3)	
O1	0.0403 (3)	0.93783 (6)	0.1944 (2)	0.0343 (3)	
C8	0.3807 (3)	1.03227 (8)	0.2246 (3)	0.0233 (3)	
C11	0.6365 (3)	0.98760 (8)	0.6577 (3)	0.0257 (3)	
H11	0.7155	0.9713	0.8075	0.031*	
C10	0.6293 (3)	1.06125 (8)	0.3675 (3)	0.0235 (3)	
C7	0.2488 (4)	0.97246 (8)	0.3022 (3)	0.0242 (3)	
C1	0.4678 (3)	0.83113 (8)	0.6455 (3)	0.0250 (3)	
C6	0.2764 (4)	0.89454 (8)	0.6453 (3)	0.0289 (4)	
H6A	0.0764	0.8842	0.5626	0.035*	
H6B	0.2708	0.9066	0.8050	0.035*	
C2	0.6726 (4)	0.81124 (9)	0.8437 (3)	0.0298 (4)	
H2	0.6926	0.8360	0.9825	0.036*	
C5	0.4487 (4)	0.79196 (8)	0.4448 (3)	0.0322 (4)	
H5	0.3130	0.8036	0.3087	0.039*	
C4	0.6333 (4)	0.73540 (8)	0.4486 (3)	0.0351 (4)	
H4	0.6181	0.7095	0.3125	0.042*	
C9	0.3281 (4)	1.07419 (8)	0.0210 (3)	0.0286 (3)	
H9	0.1735	1.0677	−0.1078	0.034*	
C3	0.8469 (4)	0.75365 (9)	0.8304 (3)	0.0332 (4)	
H3	0.9827	0.7404	0.9646	0.040*	
H3N	0.869 (4)	1.1429 (12)	0.304 (4)	0.060 (7)*	

### Atomic displacement parameters (Å^2^)

**Table:**

	*U*^11^	*U*^22^	*U*^33^	*U*^12^	*U*^13^	*U*^23^
N2	0.0245 (7)	0.0290 (7)	0.0283 (7)	0.0012 (6)	0.0011 (6)	−0.0026 (6)
N1	0.0251 (6)	0.0209 (6)	0.0264 (7)	0.0009 (5)	0.0052 (5)	0.0002 (5)
N3	0.0274 (7)	0.0252 (7)	0.0290 (8)	−0.0025 (6)	0.0042 (6)	−0.0006 (6)
N5	0.0329 (8)	0.0251 (7)	0.0411 (9)	0.0029 (6)	0.0108 (7)	0.0017 (6)
N4	0.0343 (8)	0.0293 (7)	0.0288 (8)	0.0020 (6)	0.0045 (6)	0.0010 (6)
O1	0.0323 (6)	0.0308 (6)	0.0355 (7)	−0.0077 (5)	−0.0025 (5)	−0.0025 (5)
C8	0.0213 (7)	0.0222 (7)	0.0252 (8)	0.0027 (6)	0.0020 (6)	−0.0028 (6)
C11	0.0259 (8)	0.0267 (8)	0.0222 (8)	0.0053 (6)	0.0003 (6)	−0.0015 (6)
C10	0.0227 (7)	0.0215 (7)	0.0263 (8)	0.0017 (6)	0.0050 (6)	−0.0034 (6)
C7	0.0225 (7)	0.0223 (7)	0.0268 (8)	0.0032 (6)	0.0032 (6)	−0.0039 (6)
C1	0.0264 (8)	0.0221 (7)	0.0280 (8)	−0.0036 (6)	0.0096 (7)	0.0014 (6)
C6	0.0298 (9)	0.0261 (8)	0.0334 (10)	0.0017 (7)	0.0130 (8)	0.0010 (6)
C2	0.0357 (9)	0.0269 (8)	0.0256 (8)	−0.0006 (7)	0.0041 (7)	0.0001 (6)
C5	0.0360 (10)	0.0319 (9)	0.0264 (9)	0.0023 (7)	0.0019 (7)	−0.0006 (7)
C4	0.0448 (11)	0.0282 (9)	0.0337 (10)	0.0017 (8)	0.0114 (9)	−0.0033 (7)
C9	0.0296 (8)	0.0289 (8)	0.0252 (8)	0.0009 (7)	0.0015 (7)	−0.0023 (6)
C3	0.0317 (9)	0.0289 (8)	0.0360 (11)	−0.0002 (7)	0.0006 (8)	0.0058 (7)

### Geometric parameters (Å, °)

**Table:**

N2—C11	1.293 (2)	C8—C7	1.425 (2)
N2—C10	1.366 (2)	C11—H11	0.9300
N1—C11	1.379 (2)	C1—C5	1.384 (2)
N1—C7	1.415 (2)	C1—C2	1.386 (2)
N1—C6	1.4725 (19)	C1—C6	1.510 (2)
N3—C10	1.339 (2)	C6—H6A	0.9700
N3—N4	1.363 (2)	C6—H6B	0.9700
N3—H3N	0.897 (16)	C2—C3	1.385 (3)
N5—C3	1.325 (2)	C2—H2	0.9300
N5—C4	1.336 (2)	C5—C4	1.383 (2)
N4—C9	1.324 (2)	C5—H5	0.9300
O1—C7	1.231 (2)	C4—H4	0.9300
C8—C10	1.387 (2)	C9—H9	0.9300
C8—C9	1.418 (2)	C3—H3	0.9300
			
C11—N2—C10	112.37 (14)	C2—C1—C6	121.35 (15)
C11—N1—C7	123.10 (13)	N1—C6—C1	111.23 (11)
C11—N1—C6	117.67 (14)	N1—C6—H6A	109.4
C7—N1—C6	119.20 (13)	C1—C6—H6A	109.4
C10—N3—N4	111.39 (14)	N1—C6—H6B	109.4
C10—N3—H3N	126.6 (15)	C1—C6—H6B	109.4
N4—N3—H3N	122.0 (15)	H6A—C6—H6B	108.0
C3—N5—C4	117.08 (15)	C3—C2—C1	118.49 (16)
C9—N4—N3	105.96 (13)	C3—C2—H2	120.8
C10—C8—C9	104.34 (14)	C1—C2—H2	120.8
C10—C8—C7	119.42 (14)	C4—C5—C1	119.39 (16)
C9—C8—C7	136.22 (15)	C4—C5—H5	120.3
N2—C11—N1	126.12 (15)	C1—C5—H5	120.3
N2—C11—H11	116.9	N5—C4—C5	123.05 (16)
N1—C11—H11	116.9	N5—C4—H4	118.5
N3—C10—N2	125.17 (14)	C5—C4—H4	118.5
N3—C10—C8	107.61 (13)	N4—C9—C8	110.70 (15)
N2—C10—C8	127.23 (14)	N4—C9—H9	124.7
O1—C7—N1	120.18 (14)	C8—C9—H9	124.7
O1—C7—C8	128.22 (16)	N5—C3—C2	124.12 (17)
N1—C7—C8	111.59 (14)	N5—C3—H3	117.9
C5—C1—C2	117.87 (15)	C2—C3—H3	117.9
C5—C1—C6	120.77 (15)		

### Hydrogen-bond geometry (Å, °)

**Table:**

*D*—H···*A*	*D*—H	H···*A*	*D*···*A*	*D*—H···*A*
N3—H3N···N5^i^	0.90 (2)	1.96 (2)	2.840 (2)	168 (2)
C4—H4···N4^ii^	0.93	2.61	3.526 (2)	167
C9—H9···N2^iii^	0.93	2.36	3.289 (2)	174
C11—H11···O1^iv^	0.93	2.52	3.430 (2)	167

Symmetry codes: (i) −*x*+2, *y*+1/2, −*z*+1; (ii) −*x*+1, *y*−1/2, −*z*; (iii) *x*−1, *y*, *z*−1; (iv) *x*+1, *y*, *z*+1.

## References

[bb1] Ali, T. E. S. (2009). *Eur. J. Med. Chem.* **44**, 4385–4392.

[bb2] Al Subari, A., Bouhfid, R., Zouihri, H., Essassi, E. M. & Ng, S. W. (2010). *Acta Cryst.* E**66**, o454. 10.1107/S1600536810002576 PMC2979849 21579869

[bb3] Bruker (2005). *APEX2* and *SAINT* Bruker AXS Inc., Madison, Wisconsin, USA.

[bb4] Kim, D. K., Ryu, D. H., Lee, N., Lee, J. Y., Kim, J. S., Lee, S., Choi, J. Y., Ru, J. H., Kim, N. H., Im, G. J., Choi, W. S. & Kim, T. K. (2001). *Bioorg. Med. Chem.* **9**, 1895–1899. 10.1016/s0968-0896(01)00095-5 11425592

[bb5] Sheldrick, G. M. (2008). *Acta Cryst.* A**64**, 112–122. 10.1107/S0108767307043930 18156677

[bb6] Spek, A. L. (2009). *Acta Cryst.* D**65**, 148–155. 10.1107/S090744490804362X PMC2631630 19171970

[bb7] Westrip, S. P. (2010). *J. Appl. Cryst.* **43**, 920–925.

